# Examining Nonverbal Communication in Dyadic Interactions With Virtual Humans Using an Integrated Coding System: Mixed Methods Analysis

**DOI:** 10.2196/59328

**Published:** 2025-08-07

**Authors:** Analay Perez, Rae Sakakibara, Srikar Baireddy, Michael D Fetters, Timothy C Guetterman

**Affiliations:** 1 Department of Family Medicine, University of Michigan, 1018 Fuller St, Ann Arbor, MI, 48104, United States, 1 734-998-7124

**Keywords:** nonverbal communication, nonverbal behaviors, mixed methods analysis, joint display, patient-physician dyad, patient-doctor, patient-physician, dyad, dyadic, interaction, simulation, virtual human, quantitative, qualitative, secondary analysis, non-verbal

## Abstract

**Background:**

The patient-physician dyad involves both verbal and nonverbal communication. Traditional methods use quantitative or qualitative coding when analyzing dyadic data of nonverbal communication. Quantitative coding methods can capture the frequency of nonverbal communication, while qualitative coding methods can provide descriptive information on the context and nuance of the nonverbal communication expressed. Yet, limited research has examined the integration of quantitative and qualitative coding methods of nonverbal communication between a patient-physician dyad through video recordings.

**Objective:**

The objective of this formative study was to demonstrate how nonverbal communication data can be analyzed using a mixed methods analysis approach and propose an integrated coding system using a subset of the original dataset.

**Methods:**

A secondary analysis was conducted from the intervention study with a sample of 32 pairs of randomly selected video recordings based on first and second interactions after receiving feedback from a virtual human. A 2-minute segment was used to code nonverbal communication, and a codebook was developed, informed by the literature and inductive qualitative approaches. For the mixed methods analysis, we purposefully selected 2 participants from the sample of 32 who demonstrated high frequency in quantitative and qualitative coding of nonverbal behaviors. We developed a joint display to visually represent the integration of quantitative and qualitative coding methods and developed person-level meta-inferences.

**Results:**

This formative study demonstrates an approach to nonverbal communication analysis that mixes qualitative and quantitative methods. The mixed methods results indicated the frequency of participants’ (n=32) nonverbal behaviors increased after repeated interactions, including eyebrow raise, nodding, and smiling, in addition to the increased average duration of nonverbal behaviors across interactions. Illustrated through an in-depth example of integrated mixed methods coding of 2 participants from the sample, the integration of quantitative and qualitative data provided insights into nonverbal communication. Quantitatively, we captured the frequency of nonverbal behaviors while qualitatively expanding on the context for nonverbal behaviors and generating person-level meta-inferences. The joint display informed our integrated coding system for mixed methods analysis of nonverbal communication.

**Conclusions:**

The resultant integrated coding system may be helpful to researchers engaging in nonverbal communication data of dyads by providing a step-by-step method using a mixed methods analysis approach. This approach can help us to advance methods for analyzing nonverbal communication to enhance the patient-physician dyad and education on nonverbal communication. We encourage applying the integrated coding system across several subdisciplines in health sciences research to identify how it can be further expanded and refined.

## Introduction

### Background

Verbal and nonverbal communication are important characteristics of the patient-physician dyad. Verbal communication can include but is not limited to language, dialects, and the tone of voice, whereas nonverbal communication includes eye contact, gaze gesture, facial expressions, movement, stance, body position, and spatial distancing [1,2]. These behaviors are only a subset of verbal and nonverbal behaviors included in communication. Researchers have also identified more specific nonverbal behaviors including emotions, embodiment, and interpersonal perception [3]. In clinical practice, it is vital for physicians to effectively communicate with patients using verbal and nonverbal behaviors to improve health outcomes and the overall patient-physician dyadic encounter [4]. Physicians who can appropriately interpret patients’ nonverbal cues lead to increased patient satisfaction, and patients are more likely to return for follow-up visits than physicians who are less likely to read patients’ nonverbal cues correctly [5]. Appropriate use of nonverbal cues between a patient and a physician can contribute to a positive visit, increase patient self-disclosure, understanding, and recall of information discussed during the visit, and influence a patient’s adherence to a physician’s recommendations [6]. Not only is verbal and nonverbal communication critical to the patient-physician dyad, but nonverbal communication is pivotal for accreditation of residency programs and physician certifications as these assess physicians’ competency in interpersonal skills, which involve verbal and nonverbal communication [7]. For example, the Accreditation Council for Graduate Medical Education includes a core competency of interpersonal and communication skills comprising both verbal and nonverbal communication. Although a wealth of literature exists on the benefits of enhancing verbal and nonverbal communication in health sciences research and the types of gestures commonly associated with each type, additional methodological guidance is needed when coding, analyzing, and integrating verbal and nonverbal using a mixed methods approach. Hillen et al [8] conducted a systematic review synthesizing quantitative and qualitative coding methods for assessing the interaction between health care professionals and patients and/or caregivers. However, none of the articles included in their review used mixed methods research, that is, the intentional integration of quantitative and qualitative research to better understand the research phenomenon of health care professionals and patient and/or caregiver interactions. Therefore, research is needed to understand the potential of using mixed methods analysis to holistically explore verbal and nonverbal communication.

### Traditional Methods for Analyzing Nonverbal Communication

Traditionally, both quantitative and qualitative methods are used to analyze nonverbal communication. Coding is a process of identifying segments of data, such as a brief video snippet, and applying a code as a label for what is occurring. From a quantitative perspective, the most common method to code and analyze facial expressions involves manual coding of specific facial expressions and movements using frequencies [9]. The Facial Affect Coding System (FACS) [10,11] has been widely used by several researchers and has demonstrated strong psychometric properties [12]. The FACS was created to identify facial expressions, including details on their intensity and timing. Trained coders are directed to code all possible facial expressions into action units, which are based on underlying muscle movements mapped to specific and observable facial expressions. However, 2 limitations of the FACS are that it does not account for body language and only focuses on one person rather than the interactions.

Another common approach to analyzing nonverbal behaviors is using quantitative content analysis. This process includes developing an established codebook based on manifest behavior codes and recurring codes to guide coders’ decisions [13]. Quantitative content analysis uses a similar approach as the FACS; however, it is less structured and based on an established codebook developed by the research team. Both approaches use frequencies to determine the total number of specified behaviors expressed by each participant. In either case, researchers can conduct statistical analyses if a large enough sample size exists. Though quantitative methods for coding nonverbal communication can help quantify the frequency of specific behaviors, they fail to provide context for how or why individuals may be expressing those behaviors.

Qualitatively, researchers can expand on the quantitative codes by exploring additional qualitative descriptors of each nonverbal communication. Qualitative content analysis can be implemented by developing codes and themes using an inductive process. In other words, researchers do not begin with a pre-established codebook but let the data drive what codes they develop and identify. Quantitative content analysis can help uncover what common nonverbal behaviors are, particularly when examining the patient-physician dyad, while qualitative content analysis can help reveal why by providing additional verbal and nonverbal details to complement the quantitative coding [13].

A more specific qualitative coding method is live coding [14]. Live coding is a method that allows researchers to code both verbal and nonverbal behaviors while listening to and watching video recordings [14]. The Rotor Interaction Analysis System (RIAS) uses a variation of live coding by accounting for some nonverbal behaviors qualitatively, though it primarily uses a quantitative coding system [15]. Given the advent of digital data such as video, audio, social media, music, photographs, and films in qualitative research, and the advancements of computerized assisted qualitative data analysis software, researchers are using digital software tools to facilitate this process and simultaneously code both video and audio data. As noted by Parameswaran and colleagues [14], this allows researchers to “gather contextual information from the video clips to better situate the themes when coding.”

Nonetheless, qualitatively coding nonverbal communication fails to provide the magnitude of a particular nonverbal behavior and may be less precise than quantitative coding. Therefore, merging quantitative and qualitative coding may be an ideal solution to further deepen understanding by providing quantifiable and contextual descriptions of nonverbal communication between dyads. Yet, limited research has explored the potential benefits of integrating quantitative and qualitative coding methods using a mixed methods analysis approach to examine nonverbal communication.

### Mixed Methods Analysis of Nonverbal Communication

Mixed methods research is a methodology that intentionally integrates quantitative and qualitative research methods to obtain a holistic understanding of the research questions and objectives [16]. When integrating quantitative and qualitative coding at the methods level, a single video dataset can be analyzed using an inherently mixed methods analysis approach. Onwuegbuzie and Abrams [1] proposed several reasons for integrating quantitative and qualitative coding. One reason is to reduce the dimensionality of qualitative nonverbal communication data to subsequently further explore using quantitative analysis, such as exploratory factor analysis. Another approach for integrating quantitative and qualitative coding can be to compare quantitative and qualitative nonverbal data through a joint display to generate new conclusions called meta-inferences. Alternatively, using the same dataset, another approach is to transform quantitative nonverbal data to analyze qualitatively by exploring salient themes or link the qualitative nonverbal data with quantitized nonverbal or verbal communication data to examine its correlation. Quantitizing data refers to transforming qualitative coded data into a quantitative representation, while qualitizing data refers to transforming numerical results into qualitative codes and themes [17,18]. This process can be viewed as a mixed methods coding technique [19].

Onwuegbuzie and Abrams [1] have conducted recent research on analyzing nonverbal communication using mixed methods analysis and provided useful examples from empirical research to guide researchers. To demonstrate the applications of mixed methods research for nonverbal communication, researchers [1] present several examples of studies, most involving qualitative interviews as a method for coding nonverbal communication, as well as in-person observation coding, and using a combination of digital technologies including video recordings and photographs/field notes, with examples primarily derived from the field of education. Given the role and importance of nonverbal communication in health care and health professions education, it is imperative to explore how researchers can use mixed methods research to integrate quantitative and qualitative coding of nonverbal communication at the methods level. Thus, our aim was to develop and pilot a mixed methods approach to analyze nonverbal communication in health care.

### Research Aims

The overarching purpose of this formative study was to develop and pilot an integrated coding system for mixed methods analysis of nonverbal communication and illustrate how researchers can integrate quantitative and qualitative coding methods of nonverbal communication. Particularly, this study used data from an existing video interaction dataset of simulated health care encounters between a physician and a virtual human to explore nonverbal behaviors. For this formative study, we developed an integrated coding system using a subset of data from the original sample. The aims were to: (1) understand how nonverbal behaviors could be improved through learner interactions with a virtual human across repeated scenarios and (2) use a novel mixed methods approach to analyze quantitative and qualitative coded data of nonverbal communication to provide greater context for each nonverbal behavior exhibited by participants.

## Methods

### Participants

This analysis relied on secondary data collected as part of a larger trial [20]. Participants in this study consisted of second-year medical students recruited from 3 institutions in the United States.

### Data Sources

We conducted a secondary analysis from the intervention arm of a randomized controlled trial (n=210 medical students and 840 video recordings of interactions) of a communication simulation using virtual humans. Our sample for developing and piloting a novel mixed methods analysis of nonverbal communication consisted of n=32 pairs of randomly selected video recordings of first and second interactions after receiving feedback from the virtual human program, representing approximately 15% of the entire dataset.

The duration of interactions was typically between a range of 4 and 8 minutes; however, we used the “thin slice” method for coding, which involved coding short excerpts of the video recordings. Evidence shows that short observations of 1 to 3 minutes can represent a participant’s nonverbal behavior and predict clinical and social-psychological outcomes [21]. We chose to code 2-minute segments to account for the pauses in conversation while learners read prompts on the screen. We began coding 2 minutes into the videos because of the likelihood of capturing the interaction at a point where the learner is confronted by virtual humans in emotionally charged states after some initial exchanges. The virtual human simulation, MPathic-VR (Medical Cyberworlds), provides automated feedback after the first interaction, and the learner is given another opportunity to practice in their second interaction with the same virtual human(s). Additional information on the simulation and larger study can be found in a protocol paper [22].

### Virtual Human Scenarios and Procedures

Participants in the intervention arm interacted with the MPathic-VR and participated in two related scenarios. One scenario focused on intercultural communication between a virtual human patient, the virtual human patient’s mother, and the learner. The second scenario focused on interprofessional communication with a nurse virtual human and the learner [20]. Participants in the intervention arm received personalized feedback on their performance (both verbal and nonverbal) after each scenario and a video recording of their conversations with the virtual human.

### Quantitative Analysis

Using the established codebook, we first identified which codes would be treated quantitatively using frequencies. After the 2 analysts coded the nonverbal quantitative behaviors by counting the total number of times each behavior was expressed in the 2-minute video segment for the first and second interactions with a virtual human. We summed the number of expressions for each nonverbal behavior across all videos to produce a total number for interactions one and two. We also summed the number of nonverbal behaviors expressed by the participant across the learner and virtual human talk time. Multimedia Appendix 1 provides the codebook describing each code, its definition, and whether the code was considered a quantitative or qualitative code.

### Qualitative Analysis

We began our analysis by reviewing the literature on measures and coding schemes for analyzing nonverbal behaviors. Specifically, we included in the codebook behaviors emphasized by the MPathic-VR system (eg, smiling, nodding, and eyebrow raises), in addition to nonverbal behaviors identified as expressions that may indicate compassion as described by the FACS [23]. These included eye gaze, head orientation, forward lean, oblique eyebrows, furrowed eyebrows, lower eyelid raise, slight mouth press, and lip corner puller. Coding of these behaviors was guided by the FACS [10,11]. We used the FACS in this investigation because the action units have a specific anatomical basis, which allowed for reliable application of nonverbal behavior codes across coders.

Qualitative codes were identified using open coding of the data to generate additional codes. The 2 coders (TCG and RS) also created new codes (not part of FACS) inductively through qualitative open coding. Both were experienced and trained in thematic and content analysis, one at the PhD level and the other, a research assistant with over three years of experience with qualitative data. These qualitative codes (eg, intonation and nodding) were necessary because of their prominence in the data and because the FACS does not capture such behavior. We imported video recordings into MAXQDA 2022 qualitative software for coding [24]. The two individuals conducting the initial open coding reviewed three video segments second-by-second to compare and discuss codes through consensus and reach a final codebook. Each code was defined for consistency, and we ensured all analysts were familiar with the codebook. All authors participated in the coding and review of videos and were blinded as to whether the video was the first or second interaction. We applied the qualitative codes to the relevant segment of video, which is a time duration with a start and end point precise to a second using the MAXQDA software. Any discrepancies in coding were resolved through discussions after each video was coded.

### Ethical Considerations

The original study was approved by the University of Michigan’s Institutional Review Board (HUM00067336) as an exempt study under the educational research category. Written informed consent was waived for participants. The secondary analysis reported in this paper was also approved by the University of Michigan’s Institutional Review Board (HUM00134766). Participants did not receive compensation. It was not possible to completely anonymize the video data, but we removed all participant identifiers and assigned a random study identifier. After coding, the videos were unlinked to develop a deidentified dataset.

## Results

### Nonverbal Behaviors Demonstrated by Learners

To provide a more thorough examination of nonverbal behaviors, we first investigated whether behaviors emphasized by the MPathic-VR system and other nonverbal behaviors that may convey empathy increased after repeat scenarios by examining the frequency and duration of nonverbal behaviors across videos from 32 pairs. As demonstrated in Table 1, the frequency of nonverbal behaviors emphasized by MPathic-VR increased in the second interactions compared to the first, including eyebrow raise (T_1_=111 instances, T_2_=128 instances; here T_1_=Time 1 or first interaction and T_2_=Time 2 or second interaction), nodding (T_1_=112 instances and T_2_=211 instances), and smiling (T_1_=33 instances and T_2_=38 instances). The frequency of all other nonverbal behaviors also increased in the second interaction compared to the first, except for lower eyelid raises and furrowed eyebrows, which remained consistent across scenarios (73 instances and 53 instances, respectively). Table 2 shows that the average duration of nonverbal behaviors regarding orientation increased in the second interaction compared to the first, including the average duration of eye gaze toward the virtual human (T_1_=61.3 s and T_2_=76.3 s), forward lean (T_1_=37.5 s and T_2_=38.9 s), and head tilting (T_1_=34.2 s and T_2_=48.9 s).

**Table 1.** Frequency of nonverbal behaviors in the first and second interactions.

Nonverbal behaviors	Code frequency (instances across n=32 videos), n	
	First interaction	Second interaction
Behaviors emphasized by MPathic-VR		
Eyebrow raise	111	128
Nodding	112	211
Smiling	33	38
Other facial expressions		
Furrowed eyebrows	73	73
Lip corner puller	90	120
Lower eyelid raise	53	53
Oblique eyebrows	45	62
Slight lip press	90	115

**Table 2.** Duration of nonverbal behaviors in the first and second interactions.

Orientation	Average code duration per video (seconds)	
	First interaction	Second interaction
Eye gaze toward virtual human	61.3	76.3
Forward lean	37.5	38.9
Head tilting	34.2	48.9

**Figure 1.**
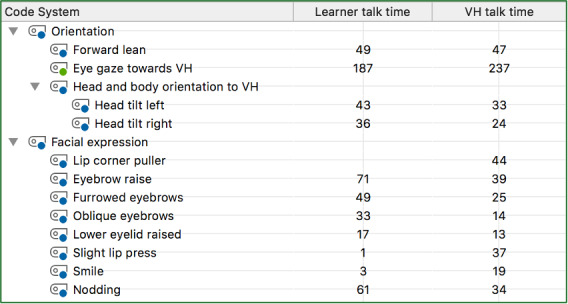
First interaction with virtual human: Frequency of simultaneous occurrences of nonverbal behavior codes when the learner or virtual human was talking during video scenario. VH: virtual human.

**Figure 2.**
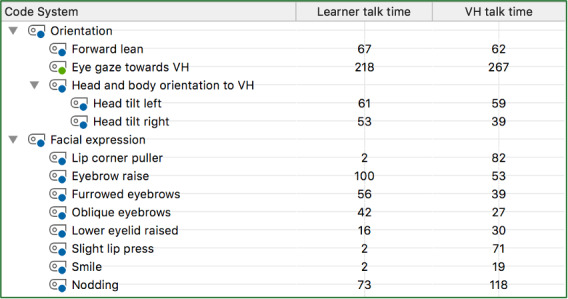
Second interaction with virtual human: Frequency of simultaneous occurrences of nonverbal behavior codes when the learner or virtual human was talking during video scenario. VH: virtual human.

We also examined whether the frequency across all nonverbal behaviors increased after repeated scenarios while either talking or listening to the virtual human (see Figures1  2). The frequency of nonverbal behaviors across learner and virtual human talk time increased from the first interactions to the second interactions, except for lower eyelid raise and smiling. The frequency of lower eyelid raise decreased between the first and second interaction for learner talk time (T_1_=17 and T_2_=16 instances) and smiling (T_1_=3 and T_2_=2 instances). The frequency of smiling was the only nonverbal behavior that did not increase after repeated scenarios during the virtual human talk time (T_1_=19 instances and T_2_=19). Figure 1 shows the nonverbal behaviors of the first interactions, and Figure 2 displays these behaviors across the second interactions. These figures are extracted from the MAXQDA software (VERBI Software) demonstrating a Code Relations Browser with the respective frequencies of each nonverbal behavior between the learner and virtual human talk time.

### Mixed Methods Analysis of Nonverbal Communication

To illustrate the application of mixed methods analysis of nonverbal communication, 2 participants were purposefully selected from the sample based on the highest frequency of quantitative and qualitative coded segments of nonverbal behaviors. To analyze the quantitative and qualitative codes and integrate the data, we found concordance between quantitative and qualitative nonverbal communication codes across segments that were close in time. Table 3 displays the joint display of integrated findings across quantitative and qualitative coded nonverbal communication data. A joint display is a visual display used to facilitate and represent the integration of the quantitative and qualitative phases in mixed methods research [25]. The joint display presented is categorized by the time segment and its respective quantitative codes, the time segment and its respective qualitative codes, and the person-level meta-inferences. Meta-inferences, or integrated conclusions from the quantitative and qualitative strands, were developed by merging quantitative and qualitative coding inferences to fully understand participants’ nonverbal communication and further augment the verbal data.

We generated meta-inferences at the person-level by aggregating quantitative and qualitative findings across all time points for each person to generate a rich description of each participant’s overall nonverbal communication (see Table 3 for person-level meta-inferences). For example, participant 047 for 2:06-2:30 engaged in forward lean while at the same time nodding their head upwards and downwards. When integrating these results within the context of the video scenario, we can conclude the participant was engaged in the conversation and displayed nonverbal behaviors consistent with the verbal data, affirming that they knew what was best for the patient. Collectively, these were integrated to generate a person-level meta-inference representing the nonverbal communication displayed by each participant for the entire time segment.

Table 4 displays the frequencies for each quantitative nonverbal behavior code across each participant. Compared to other quantitative nonverbal codes, participant ID 047 engaged in forward lean and learner talk time at the highest frequency (four and three instances, respectively). Participant ID 057 displayed eye gaze toward the virtual human at the highest frequency (7 instances), followed by learner talk time and virtual human talk time (3 instances, respectively). These results help to further contextualize the qualitative findings from the joint display by providing quantifiable data across nonverbal behaviors expressed by the participants.

**Table 3.** Joint display of integrated nonverbal coding analysis and person-level meta-inferences.

Time		Quantitative codes	Time	Qualitative codes	Person-level meta-inference
ID 047					
2:06-2:30		Forward lean	2:09-2:12	Nodding head up and down	The participant was engaged in the conversation and displayed nonverbal communication in response to the virtual human regarding steps they took to alleviate tension between the patient and their mother. Participant’s nonverbal cues demonstrated that they cared about the outcome of the conversation and explained the actions they took to alleviate tension between the patient and their mother. The participant’s gaze was focused on the virtual human, and they adequately responded all questions posed by the virtual human. The participant displayed nonverbal behaviors such as shaking their head while agreeing with the virtual human’s response when learning about confidential patient information.
2:06-2:35		Forward lean and learner talk time	2:30-2:32	Shakes head side-to-side	
2:34-2:58		Forward lean and learner talk time	2:54-2:58	Shakes head	
2:55-3:48		Forward lean	3:31-3:32	Nodding head	
2:57-3:00		Eye gaze toward virtual human			
3:47-4:15		Learner talk time	3:47-3:48	Inconsistent behavior	
ID 057					
2:03-2:06		Learner talk time and eye gaze toward virtual human	2:05-2:07	Nodding head up and down	The participant focused on the virtual human’s response while nodding their head up and down to affirm patient’s autonomy. During this process, the participant nodded their head forward and backward, closed their eyes, and their lips read “okay” as if to imply that there could be an issue with the information that was disclosed about the patient. The participant nodded their head back and forth while quietly under breath stating, “I see,” based on confidential patient information disclosed by the virtual human. Then, they nodded their head and affirmed they did not understand the situation, implying the participant was admitting to making a mistake. The participant was focused on the response from the virtual human and nodded their head in a manner that demonstrated they understood the situation and closed their eyes as if to sympathize with the patient. As the virtual human continued to share information, the participant nodded their head back and forth in agreement. The participant frowned and pressed their lips to the corner of their mouth to show signs of sympathy and disbelief. The participant nodded their head forward and backward to show that they understood what was happening based on the information shared with them about the patient by the virtual human. The participant’s nonverbal behaviors aligned with their agreement to the virtual human’s comment on the importance of working as a team in health care with the patient and doctors.
2:24-2:43		Virtual human talk time and eye gaze toward virtual human	2:43-2:44	Nodding head forward and backward	
2:45-2:55		Learner talk time and eye gaze toward virtual human	2:46-2:47	Nodding head side-to-side	
2:52- 3:22		Virtual human talk time and eye gaze toward virtual human	3:01-3:23	Nodding heading, nodding head front to back, frowns, and presses lips to the corner of mouth	
3:24-3:46		Virtual human talk time and eye gaze toward virtual human	3:24- 3:29	Nodding head forward and backward	
3:47-3:51		Eye gaze toward virtual human and learner talk time	3:45-3:48	Nodding head forward and backward and inconsistent behavior	
3:57-3:59		Eye gaze toward virtual human	3:57-3:58	Nodding head forward and backward	

**Table 4.** Frequencies of quantitative nonverbal codes expressed by participants.

Quantitative code	Frequency, n	
	Participant ID 047	Participant ID 057
Forward lean	4	0
Learner talk time	3	3
Eye gaze toward virtual human	1	7
Virtual human talk time	0	3

## Discussion

### Principal Findings

The findings from this study demonstrate that the frequency of nonverbal behaviors increased after repeated scenarios between the first and second interactions. Specifically, all nonverbal behaviors emphasized by the MPathic-VR, including eyebrow raise, nodding, and smiling, increased from the first to second interactions. The average duration (in seconds) of nonverbal behaviors, including eye gaze toward virtual human, forward lean, and head tilting, also increased from the first to second interactions. Overall, these results provide some evidence of the validity of our coding approach, given the behaviors expected to increase did increase based on the nonverbal coding.

These results are particularly promising regarding patient-physician interactions. Physicians who display empathic nonverbal behaviors increase patients’ perceptions of physician empathy, warmth, and competence [26]. Furthermore, nonverbal behaviors such as patient-centered gaze and body orientation positively affect a patient’s perceived level of physician empathy [27]. Therefore, these findings hold clinical implications for training effective nonverbal communication skills among medical students and professionals in clinical practice. Furthermore, these results are supported in the literature demonstrating that students who interacted with MPathic-VR and received feedback from a simulated scenario improved their communication scores, particularly nonverbal communication [20]. The findings from this study used the same virtual human, the MPathic-VR, and reported the benefits of using virtual humans as it allowed for a standardized method for comparison across participants [20]. Further research is needed to ensure the findings from a virtual human are replicated in real-world settings.

Through this formative study, we highlight the role of using video recordings and mixed methods analysis to obtain a more comprehensive understanding of how learners improve their nonverbal communication through virtual human video interactions. Using video rather than photos has been shown to be a more ecologically robust method for analyzing nonverbal communication [26]. From a mixed methods analysis perspective, this study sheds light on a novel approach by coding quantitative and qualitative nonverbal data to generate a more holistic understanding with descriptions and context for each behavior. By analyzing and integrating quantitative and qualitative coding of nonverbal behaviors, we generated meta-inferences that illuminated more nuance of each nonverbal behavior displayed by 2 participants from the sample. As such, one of the contributions of this study was the development of a step-by-step approach elucidating an integrated coding system for mixed methods analysis of nonverbal communication and strategies to guide researchers in analyzing nonverbal communication data. Below we provide a step-by-step approach for an integrated coding system:

First, determine the duration of the coded segment: The first step is to identify the duration of the video that will be coded. As noted, we used the thin slice method, but if there is a rationale for a longer duration, it is important to justify the length of time that will be coded. It is also critical to identify whether coding will occur toward the beginning, middle, or end of the video, explicitly outlining the start and end times for each clip.

Second, developing a codebook of nonverbal behaviors: Afterwards, it is important to identify the quantitative and qualitative nonverbal codes that will be incorporated into the codebook. These codes may be informed by literature, theory, or the study. Quantitative codes involve using frequencies or counts to document nonverbal behaviors such as eye gaze, head tilt, and smile. Qualitative codes can provide further details of each behavior. For example, if a qualitative code is based on positive or negative intonation, researchers may expand on this code by describing whether participants appeared uninterested during the interaction, the context of the discussion, and the point at which the participant starts to show signs of disinterest. When developing a codebook, it is encouraged to collaborate with all members of the research team who will be engaging in the coding and data analysis phase to ensure consensus on the codebook, determine distinctions between quantitative and qualitative nonverbal codes, and ensure each code is operationalized to increase reliability among coders.

Third, the coding process: Determine how many individuals from the research team will be involved in the coding process, how many video clips each individual on the research team will code, the number of times individuals will meet to discuss codes, and strategies to ensure the validity and reliability of the data throughout the coding process. The team should also decide whether software such as MAXQDA, NVivo (QSR International), or any other qualitative software programs will be used to code video data or whether other alternatives, such as Microsoft Excel, will be used to organize coded information.

Fourth, integrating quantitative and qualitative coded data of nonverbal behaviors: Once all videos have been coded quantitatively and qualitatively, researchers will be tasked with determining the most appropriate ways to integrate the data. Given that nonverbal communication data can be analyzed quantitatively and qualitatively, we encourage researchers to use mixed methods analysis approaches [1]. When using mixed methods analysis to analyze nonverbal data, consider the purpose of conducting a mixed methods analysis. These can include but are not limited to reducing data, using a joint display, data transformation, correlating data, merging verbal with nonverbal data to generate new codes or variables, comparing data, confirming data, or using findings based on the qualitative coded data of nonverbal behavior to inform the quantitative analysis [1]. Determining the reason for conducting a mixed methods analysis of nonverbal data will depend upon the research questions and the study’s objectives. Thus, researchers are encouraged to revisit their research questions and aims frequently to determine the most adequate approach for the mixed methods analysis of nonverbal data.

Fifth, presenting a visual display and developing meta-inferences: To visually depict the integration of quantitative and qualitative coding of nonverbal data and findings, a joint display can be used to represent this information and aid in generating meta-inferences. A joint display can be used to integrate qualitative and quantitative data, methods, or results using a visual representation [25]. To identify meta-inferences, a researcher can match verbal and nonverbal domains to get a better understanding of the phenomenon. For example, if integrating the quantitative and qualitative data using matching, a researcher may code the type of verbal interactions and the context of verbal responses with the corresponding nonverbal behaviors to identify the meta-inferences. When developing meta-inferences, the context of the interaction will be particularly helpful. Analysts familiar with the coding scheme should work together to develop these meta-inferences. As a method of member check, researchers may share the developed meta-inferences with participants to ensure the validity of the findings. This will allow for participants to provide feedback, revisions, and additions to the proposed meta-inferences. We encourage researchers to use creativity to explore the best strategies to visually represent the data and findings, reinforcing thoughtful consideration of the generated meta-inferences.

### Limitations

This study has several limitations. First, the nonverbal analysis is based on a small, formative probabilistic sample of only 32 individuals. Results are not likely generalizable, as the goal was to develop methods of nonverbal analysis. A second limitation of this study is that data in the joint display are only displayed for 2 participants. However, we purposefully selected these participants to demonstrate how data can be integrated and analyzed using mixed methods research. Future research can incorporate more participant examples in a joint display to further highlight nuances of integration and analysis of quantitative and qualitative coding of nonverbal behaviors. Furthermore, we present and code several nonverbal behaviors, but we also recognize that this is not an exhaustive list of all nonverbal behaviors present during a patient-physician interaction. In addition, although we provided a codebook and defined each nonverbal behavior, there may be issues of coder drift. However, we aimed to capture relevant nonverbal behaviors in healthcare that appear related to empathic communication and explained these behaviors in the codebook to ensure behaviors were coded consistently. Yet, linking specific behaviors to emotions or underlying intent is tenuous and beyond the scope of this formative study. We encourage researchers to expand on these nonverbal codes by examining the literature in their respective fields to determine additional codes that can be incorporated.

### Conclusions

This study aimed to develop and pilot a mixed methods coding system to analyze verbal and nonverbal behaviors using video data to obtain a more comprehensive understanding of nonverbal behaviors. Although nonverbal communication may contradict verbal communication, being cognizant of nonverbal behaviors can help physicians better express their nonverbal communication responses [4]. As a result, enhancing nonverbal communication skills in health settings can help to build trust, foster healing, and improve health outcomes [4]. Furthermore, we demonstrated how using a mixed methods analysis approach to code nonverbal communication can be particularly beneficial when analyzing video data of patient-physician communication to expand on both verbal and nonverbal behaviors exhibited by each individual. Using mixed methods analysis, we developed a novel approach to analyzing nonverbal communication that builds on existing literature by providing a formative demonstration and proposing an integrated coding system. We aim for this integrated coding system to shed light for researchers analyzing nonverbal communication data across various subdisciplines within health sciences research. We encourage researchers to investigate creative paths for analyzing nonverbal communication data using video recordings through a mixed methods analysis approach that advances discussions on enhancing the patient-physician dyad across multiple clinical settings.

## Supplementary material

10.2196/59328 Multimedia Appendix 1 Codebook excerpt derived from Facial Affect Coding System (FACS) and inductive qualitative coding.

## Abbreviations

FACS: Facial Affect Coding System
RIAS: Rotor Interaction Analysis System
