# Supplementary material for: Examining Nonverbal Communication in Dyadic Interactions With Virtual Humans Using an Integrated Coding System: Mixed Methods Analysis
Source: JMIR Form Res. 2025 Aug 7;9:e59328. doi: 10.2196/59328 (PMC12331217; doi:10.2196/59328)
Supplement: Multimedia Appendix 1 [file formative-v9-e59328-s001.docx]

**Appendix 1**

**Table 1**

Excerpt of Codebook Derived from FACS and Inductive Qualitative Coding

| **Code** | | | **Definition** | **Quantitative or Qualitative Code** | **Format for Assessing Behavior** |
| --- | --- | --- | --- | --- | --- |
| **Orientation** | | | | |  |
|  | Forward lean | | Instances where learner leans in towards the camera, usually can see shoulders coming forward.  NOTE: this movement is different from just the head tilting forward. | Quantitative | Frequency |
|  | Eye gaze towards virtual human | | Time the learner is directing gaze at virtual human. NOT coded when learner is looking at prompts | Quantitative | Frequency |
|  | Head and body orientation to virtual human | | Head up  Head down  Head forward  Head back | Quantitative | Frequency |
|  |  | Head tilt left | Tilt left | Quantitative | Frequency |
|  |  | Head tilt right | Tilt right | Quantitative | Frequency |
| **Facial expression** | | | | |  |
|  | Lip corner puller | |  | Quantitative | Frequency |
|  | Eyebrow raise | | Not in the literature we reviewed as facial actions conveying empathy, but because we saw eyebrow raising movements that were not applicable to the oblique eyebrows code, we decided to add this code.  NOTE: where intensity in eyebrow movement is lessened during a segment but brows don’t return back to neutral, that portion is continuously coded through until brows return to baseline (in other words, coded segments may have a variety of brow raising intensities). | Quantitative | Frequency |
|  | Furrowed eyebrows | | Brow lowered  Brows lowered and/or drawn together. Wrinkles appear just between brows | Quantitative | Frequency |
|  | Oblique eyebrows | | Inner brow raise + Brow lowered  Inner corner of the brow lifted and pulled together resulting in an oblique shape or dip in center and up at inner corner. Outer corner of eyebrow is not lifted, and if oblique shape is present, will be somewhat lower than in neutral.  NOTE: where intensity in eyebrow movement is lessened during a segment but brows don’t return to neutral, that portion is continuously coded through until brows return to baseline (in other words, coded segments may have a variety of brow raising intensities). | Quantitative | Frequency |
|  | Lower eyelid raised | | Lid tightener  Lid tightener (both upper and lower lid) | Quantitative | Frequency |
|  | Slight lip press | | Lip presser  Presses the lips together without pushing up the chin boss. Lowers the upper lip and raises the lower lip to a small extent. Tightens and narrows the lips-- lips appear de-elongated due to the narrowing and pressing of the center parts of the lips. May cause a bulging of the skin above the upper lip and/or below the lower lip | Quantitative | Frequency |
|  | Smile | | Lip corner puller and Cheek raised  Pulls the corners of the lips back and upward. Deepens the nasolabial furrow, pulling it laterally and up. The skin adjacent to the nasolabial furrow is raised up and laterally.  Raises the infraorbital triangle, lifting the cheek upwards. May cause crow's feet lines or wrinkles to appear, extending radially from the outer corners of the eye aperture.  Distinguish smile from a slight lip press by looking at upper face engagement (cheek raised, eyelid movement, etc.) | Quantitative | Frequency |
| **Others** | | | | |  |
|  | Nodding | | Instances of listener feedback, displayed through cyclical or continuous, upward/downward, or forward/backward motions of the vertical or sagittal plane. Includes instances of speaker nodding to add emphasis to what is said based on the context of the conversation.  Includes head shaking | Qualitative and Quantitative | Content analysis and frequency |
|  | Learner talk time | | Time learner is speaking | Quantitative | Frequency |
|  | Virtual human talk time | | Time virtual human is speaking | Quantitative | Frequency |
|  | Inconsistent behavior | | Verbal and nonverbal behavior do not match (i.e. shaking head while agreeing with conversation partner, smiling while talking about serious sensitive matters, etc.) | Qualitative | Content analysis |
| **Intonation** | | | | |  |
|  |  | Positive intonation | Tone of voice comes off to listener as warm, empathic, caring, attentive, and genuine | Qualitative | Content analysis |
|  |  | Negative intonation | Tone of voice comes off to listener as snarky, contemptuous, disingenuous, or uninterested | Qualitative | Content analysis |

*Note.* We used this coding scheme across the two-minute video segment.
